# Genetic diversity and population structure of *Miscanthus lutarioriparius*, an endemic plant of China

**DOI:** 10.1371/journal.pone.0211471

**Published:** 2019-02-01

**Authors:** Sai Yang, Shuai Xue, Weiwei Kang, Zhuxi Qian, Zili Yi

**Affiliations:** 1 College of Bioscience & Biotechnology, Hunan Agricultural University, Changsha, Hunan, China; 2 Orient Science & Technology College of Hunan Agricultural University, Changsha, Hunan, China; National Cheng Kung University, TAIWAN

## Abstract

*Miscanthus lutarioriparius* is a native perennial *Miscanthus* species of China, which is currently used as raw material of papermaking and bioenergy crop. It also has been considered as a promising eco-bioindustrial plant, which can offer raw material and gene for the biomass industry. However, lack of germplasm resources and genetic diversity information of *M*. *lutarioriparius* have become the bottleneck that prevents the stable and further development of the biomass industry. In the present study, genetic diversity of 153 *M*. *lutarioriparius* individuals nine populations was studied using 27 Start Codon Targeted (SCoT) markers. High polymorphic bands (97.67%), polymorphic information content (0.26) and allele number (1.88) showed SCoT as a reliable marker system for genetic analysis in *M*. *lutarioriparius*. At the species, the percentage of polymorphic loci [*PPL*] was 97.2%, Nei’s gene diversity [*H*] was 0.36, Shannon index [*I*] was 0.54 and Expected Heterozygosity [*He*] was 0.56. Genetic variation within populations (84.91%) was higher than among populations (15.09%) based on analysis of molecular variance (AMOVA). Moderate level of genetic differentiation was found in *M*. *lutarioriparius* populations (*F*_st_ = 0.15), which is further confirmed by STRUCTURE, principal coordinates analysis (PCoA) and an unweighted pair group method with arithmetic mean (UPGMA) analysis that could reveal a clear separation between groups of the north and south of Yangtze River. The gene flow of the populations within the respective south and north of Yangtze River area was higher, but lower between the areas. There was no obvious correlation between genetic distance and geographic distance. The breeding systems, geographical isolation and fragmented habitat of *M*. *lutarioriparius* may be due to the high level of genetic diversity, moderate genetic differentiation, and the population, structure. The study further suggests some measure for conservation of genetic resources and provides the genetic basis for improving the efficiency of breeding based on the results of diversity analysis.

## Introduction

*Miscanthus* spp. is a perennial herbaceous grass belonging to the Poaceae [1] with origins in East and Southeast Asia. China is the distribution center of the world’s *Miscanthus* resources, and *Miscanthus lutarioriparius* is native to China among all *Miscanthus* species [1–3]. *Miscanthus* has a strong ability to adapt to different habitats owing to rich morphological and genetic diversity [2–5]. It is considered one of the most promising second-generation energy crops with high C4 photosynthetic efficiency [6] high and stable yield potential [7,8], fast growth, low-nutrient requirement [9], high water-use efficiency [10,11], high disease resistance [12], and high cellulose content [13]. Additionally, *Miscanthus* can also provide raw materials for biorefineries to produce various chemicals, fuel, and biomaterials [14–17] and is beneficial for reducing the risk of soil erosion [18] and increasing soil carbon content and biodiversity [19]. Therefore, identification of *Miscanthus* varieties with high biomass yield and good energy-related quality is desirable.

At present, *M*. × *giganteus* is the most commonly researched and utilized miscanthus plant in both Europe and America, and is a natural triploid hybrid found from northeast Asia [10, 11, 20–22]. *Miscanthus sinensis* is the most widespread species across the world, and it has elicited great interest over its genetic diversity [23–25], ecological speciation [26], biomass yield and quality [27], conversion efficiency [28, 29], stress resistance [30, 31], effects on soil microorganisms [32] and more. There are few studies on *M*. *floridulus* and *M*. *sacchariflora*. In China, *M*. *lutarioriparius* is the only commercially used miscanthus species, which is grown for papermaking industry since 1950s [33]. Almost all the used *M*. *lutarioriparius* biomass is produced from 1.2×10^5^ ha wildly grown *M*. *lutarioriparius* in the coastal area of Dongting Lake. Therefore, its biomass yield and quality have been a historical concern. *Miscanthus lutarioriparius* is mainly distributed in the tidal flats of lakes and rivers alongside the middle and lower reaches of the Yangtze River in China [33,34].

However, due to the downturn in the paper industry, the future of *M*. *lutarioriparius* is uncertain. Fortunately, Yan *et al*. have found that *M*. *lutarioriparius* grow well in marginal lands of semi-arid and arid areas [35] and can be used to produce biomethane as an energy crop [36], which will provide a novel approach for the high value utilization of *M*. *lutarioriparius*. There are many types and large area of marginal land (769.37×10^4^ ha) available for producing miscanthus in China [37], which covers a vast area, passes multiple climate strips and has many types soils. At present, our work centers on breeding miscanthus suitable for growing in marginal areas. Collection and researched on genetic basis of miscanthus germplasm were the work key point of miscanthus breeding.

Limited information exists regarding genetic analysis of *M*. *lutarioriparius*. Therefore, it is difficult to draw any conclusion about its genetic diversity, resource protection, hybridization breeding, or screening for excellent germplasm. However, some data on genetic diversity and population structure of other *Miscanthus* species using molecular markers have been reported. Simple Sequence Repeats (SSR) [23], Sequence-Related Amplified Polymorphisms (SRAP) [38], Amplified Fragment Length Polymorphism (AFLP) [39], Inter-Simple Sequence Repeats (ISSR) [40] and Single Nucleotide Polymorphisms (SNP) [41] have been utilized in the analyses of genetic diversity of *M*. *sinensis*. In other studies, the genetic diversity and population structure of *M*. *saccharilorus* was assessed based on SSR [42], and SNP [43] markers. Tang [44] estimated genetic diversity and population structure of *M*. × *giganteus* using Diversity Arrays Technology (DArT). Therefore, molecular markers can serve an important role in unveiling the genetic diversity and population structure of *M*. *lutarioriparius*.

In recent years, a novel marker system termed start codon targeted (SCoT) markers was developed by Collard and Mackill [45] based on the short-conserved region flanking the start codon (ATG) in plant genes. SCoT employs long primers (18-mers), and can generate polymorphisms that are reproducible. It is considered as a dominant marker system, requiring no prior sequence information, and the polymorphism is correlated to functional genes and their corresponding traits. Other excellent characteristics include their simplicity of use, high polymorphism, the use of universal primers, low cost and gene targeted markers. This technique has been successfully used to assess genetic diversity and structure [46, 47], construct DNA fingerprints [48, 49], identify QTLs [50], and analyze differential gene expression and screen stress tolerance genes [51].

The present study is the first attempt to use SCoT markers to assess the level of genetic diversity of *M*. *lutarioriparius*, which were collected from the wild populations. The main objectives of this study were to assess the genetic diversity and genetic relationship of *M*. *lutarioriparius* in China. These results could benefit *M*. *lutarioriparius* germplasm collection, conservation and future breeding.

## Materials and methods

### Ethics statement

This research did not involve the rare and endangered plants. Samples of *M*. *lutarioriparius* were gathered from areas, which are not in any nature reserve or private land. Therefore, collection of the material did not require an additional approval of the ethics committee or other specific permission.

### Plant materials

One hundred and fifty-three *M*. *lutarioriparius* accessions were used in this study. The accessions were collected from 153 sites which covered the major distribution areas in China (S1 Table). Over five rhizomes from a site were sampled. Sites of accessions separated by a distance of more than 1 km were collected during the autumn and winter of 2017. The rhizomes of each accession from a site were planted in the Miscanthus Germplasm Nursery Garden (28°11'14.42"N, 113°4'7.69"E) in Hunan Agriculture University, Changsha, Hunan, China.

### Genomic DNA extraction

In the spring of 2018, young leaves from 153 plants were collected from the Miscanthus Germplasm Nursery Garden and stored at -20°C for genomic DNA isolation. Total genomic DNA was isolated from 1 g fresh young leaves of *M*. *lutarioriparius* following the method described by Doyle and Doyle [52]. The concentration and purity of DNA samples were determined by agarose gel electrophoresis analysis and spectrophotometer absorbance (Biochrom Libra S22, Biochrom Ltd., Cambridge, UK). Only DNA samples with an optical density (OD) at 260 nm/ OD at 280 nm (OD260/OD280) >1.8 were diluted in ultrapure sterile water to 50 ng μl^-1^ and then stored at -20°C for further PCR amplification.

### SCoT-PCR amplification

A total of 36 SCoT primers developed by Collard and Mackill [45], which were produced by Shanghai Sangon Biological Engineering Technology and Service Co. Ltd., and 27 primers with clear, enlarged, and rich polymorphism bands were chosen (Table 1). PCR amplification were conducted in 15 μl volumes, containing 50 ng genomic template DNA, 1× PCR buffer (100 mM Tris–HCl, 100 mM (NH_4_)_2_SO_4_, 100 mM KCl, 1% Triton X-100, pH 8.8), 2.5 mM Mg^2+^, 0.4 mM dNTPs, 0.5 μM primer, and 1 U Taq DNA polymerase (Sangon Bio., Shanghai, China). The PCR amplifications were performed using a professional thermocycler (Biometra Germany) with the following program: 94°C for 5 min, followed by 35 cycles of 94°C for 50 s, 55.3–64.1°C for 60 s, and 72°C for 90 s, and a final extension at 72°C for 7 min. PCR products were separated on 1.5% agarose gels and stained with ethidium bromide and photographed under UV light. The amplification products generated by SCoT primers were scored as present (1) or absent (0). Only clear and repeatable bands were considered.

**Table 1:** SCoT primers used for this study and the extent of polymorphism.

Primer name	Primer sequence (5’-3’)	TNB^a^	NPB^b^	PPB^c^ (%)	PIC^d^
**ST2**	CAACAATGGCTACCACCC	14	13	92.86%	0.22
**ST3**	CAACAATGGCTACCACCG	17	17	100.00%	0.29
**ST4**	CAACAATGGCTACCACCT	11	10	90.91%	0.24
**ST5**	CAACAATGGCTACCACGA	15	15	100.00%	0.27
**ST6**	CAACAATGGCTACCACGC	19	18	94.74%	0.27
**ST7**	CAACAATGGCTACCACGG	20	19	95.00%	0.28
**ST9**	CAACAATGGCTACCAGCA	13	13	100.00%	0.25
**ST11**	AAGCAATGGCTACCACCA	17	17	100.00%	0.26
**ST12**	ACGACATGGCGACCAACG	14	14	100.00%	0.25
**ST13**	ACGACATGGCGACCATCG	13	13	100.00%	0.24
**ST14**	ACGACATGGCGACCACGC	15	14	93.33%	0.25
**ST15**	ACGACATGGCGACCGCGA	9	9	100.00%	0.23
**ST17**	ACCATGGCTACCACCGAG	19	18	94.74%	0.25
**ST18**	ACCATGGCTACCACCGCC	14	14	100.00%	0.27
**ST19**	ACCATGGCTACCACCGGC	16	16	100.00%	0.24
**ST20**	ACCATGGCTACCACCGCG	16	16	100.00%	0.30
**ST21**	ACGACATGGCGACCCACA	20	19	95.00%	0.26
**ST22**	AACCATGGCTACCACCAC	18	18	100.00%	0.27
**ST23**	CACCATGGCTACCACCAG	13	12	92.31%	0.25
**ST24**	CACCATGGCTACCACCAT	20	19	95.00%	0.26
**ST26**	ACCATGGCTACCACCGTC	17	17	100.00%	0.27
**ST27**	ACCATGGCTACCACCGTG	21	21	100.00%	0.28
**ST29**	CCATGGCTACCACCGGCC	16	16	100.00%	0.29
**ST32**	CCATGGCTACCACCGCAC	21	21	100.00%	0.28
**ST34**	ACCATGGCTACCACCGCA	13	13	100.00%	0.22
**ST35**	CATGGCTACCACCGGCCC	9	8	88.89%	0.24
**ST36**	GCAACAATGGCTACCACC	19	19	100.00%	0.29
**Mean**		15.89	15.52	97.67%	0.26
**Total**		429	419		

^a^TNP: total number of bands.

^b^NPB: number of polymorphic bands

^c^PPB: percentage of polymorphic bands

^d^PIC: polymorphism information content

### Data analysis

#### Genetic diversity

Excel 2013 was used to calculate the total number of bands (TNB), the number of polymorphic bands (NPB), and the percentage of polymorphic bands (PPB). The polymorphism information content (PIC) of SCoT primers was determined using POWERMARKER v3.25 [53]. The software POPGENE V1.32 was used to estimate the level of genetic diversity, with five parameters: observed number of alleles (*Na*), effective number of alleles (*Ne*), Nei’s gene diversity (*H*) Shannon’s information index of Diversity (*I*), and the percentage of polymorphic loci (*PPL*). Nei's expected heterozygosity (*He*) was calculated using GenAlEx v.6.1[54].

#### Population structure

The population structure of the 153 *M*.*lutarioriparius* was analyzed in the software STRUCTURE v2.3.4 [55] using admixture model, correlated allele frequencies, and a burn-in period of 100,000 iterations, followed by 1,000,000 Markov Chain Monte Carlo (MCMC) repetitions [56]. The value of K ranged from 1 to 9, with 30 independent runs. Maximum likelihood (LnP(K)) and delta K (Δ*K*) were used to identify the optimum number of subpopulations following Evanno’s methods [56]. The structure result was analyzed in Structure Harvester v0.6.94 [57]

#### Cluster analysis

Principal coordinate analysis (PCoA) was performed using GenALEx v6.1 software to detect the genetic relationships among populations. Cluster analysis was performed using SAHN from NTSYS-pc version 2.10 with the unweighted pair group method with arithmetic mean (UPGMA) algorithm. SPSS 19.0 was used to calculate the correlation between the genetic distance and geographic distance matrices. The latitude and longitude of each population was replaced by the latitude and longitude of the midpoint of the population.

#### Genetic differentiation

Analysis of molecular variance (AMOVA) was performed to analyze the genetic differentiation (*F*_*st*_) using “pegas” [58]in R. The gene flow (*N*_*m*_) among populations (calculated as *N*_*m*_ = (1–*F*_*st*_) / 4 *F*_*st*_), Nei’s genetic distance, and the genetic similarity were calculated using the software POWERMARKER v3.25 [53].

## Results

### SCoT polymorphisms

Thirty-six SCoT primers were tested with three *M*. *lutarioriparius* accessions as DNA templates; all primers produced amplification products, and only primers showing clear and reproducible band patterns were selected for further analysis. Twenty-seven primers were then chosen for species identification and phylogenetic analysis. As shown in Table 1, all 27 primers used for SCoT analysis A total of 429 fragments were obtained, and 419 of the fragments were polymorphic. The number of polymorphic fragments for each SCoT primer ranged from 7 (ST15) to 21 (ST27, 32), with an average of 15.52. The percentage of polymorphic fragments was from 88.89% to 100.00%, with an average of 97.67% polymorphism. Polymorphism information content (PIC) values were 0.22 to 0.29, with an average of 0.26. The number of different alleles was 1.97 at the species (Table 2). These results indicated that a high level of polymorphism could be detected among *M*. *lutarioriparius* accessions using SCoT markers.

**Table 2:** Genetic diversity parameters for nine populations of *Miscanthus lutarioriparius*.

Population	*N*_a_^a^	*N*_e_^b^	*H*^c^	*I*^d^	*PPL*^e^	*He*^f^
**Pop 1**	1.87	1.51	0.30	0.45	86.95%	0.50
**Pop 2**	1.90	1.54	0.31	0.47	80.21%	0.51
**Pop 3**	1.92	1.59	0.35	0.51	91.61%	0.51
**Pop 4**	1.91	1.61	0.35	0.53	91.73%	0.53
**Pop 5**	1.82	1.50	0.29	0.43	81.82%	0.48
**Pop 6**	1.75	1.41	0.24	0.37	75.29%	0.45
**Pop 7**	1.91	1.50	0.31	0.47	91.14%	0.47
**Pop 8**	1.91	1.52	0.34	0.51	90.91%	0.52
**Pop 9**	1.93	1.61	0.36	0.53	93.47%	0.54
**mean**	1.88	1.53	0.32	0.47	87.01%	0.50
**Total**	1.97	1.63	0.36	0.54	97.20%	0.56

^a^*N*_*a*_: number of different allele*s*.

^b^*N*_*e*_: average number of effective alleles.

^c^*H*: Nei’s (1973) gene diversity.

^d^*I*: Shannon diversity index.

^e^*PPL*: percentage of polymorphic loci.

^f^*He*: Expected Heterozygosity

### Population genetic diversity

The percentage of polymorphic loci (*PPL*) and Nei’s gene diversity (*H*) were important parameters for measuring the level of genetic diversity [59]. In Table 2, the genetic diversity parameters of the nine populations are shown. The *H* value ranged from 0.24to 0.36, with an average value of 0.32. *PPL* ranged between 75.29% and 93.47%, with an average value of 87.01%. Population 9 (Pop 9) showed the highest level of genetic diversity among all populations (*H* = 0.36, *I* = 0.53, *PPL =* 93.47%, *He* = 0.54), while the lowest genetic diversity parameter values were found in Pop 6 (*H* = 0.24, *I =* 0.37, *PPL =* 75.29%, *He* = 0.45). The total genetic diversity of *M*. *lutarioriparius* was high across China as indicated by the indexes of *H* = 0.36, *I* = 0.54, *PPL* = 97.20%, *He* = 0.56.

### Population structure and cluster analysis

**Fig 1:**
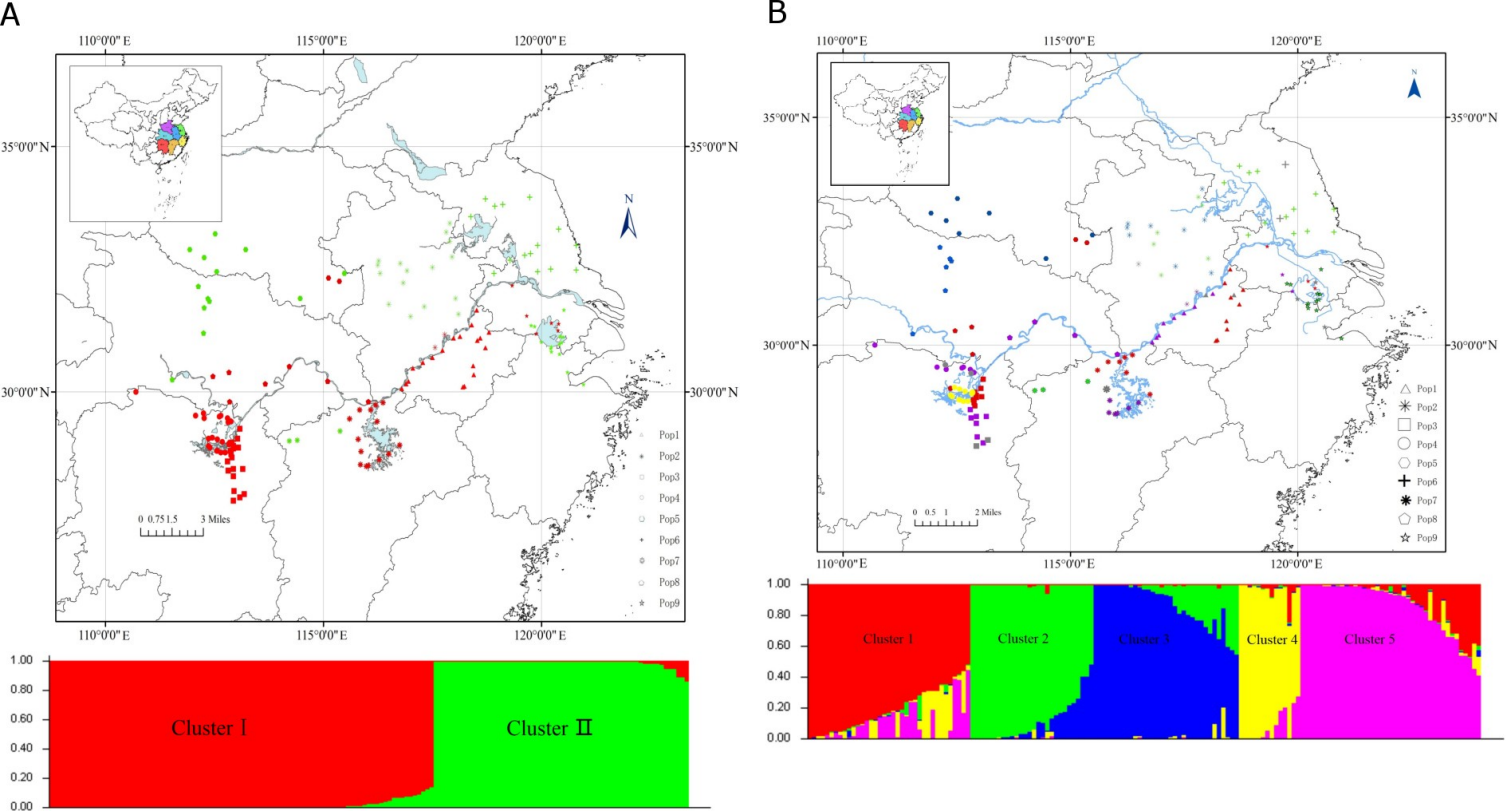
Map of nine sampled populations of and Bayesian admixture proportions identified by STRUCTURE of individual plants of *Miscanthus lutarioriparius*. (A) *K* = 2, (B) *K* = 5. Colors used in the maps are associated with STRUCTURE clusters. Grey symbols represent‘admixed’ group in the Fig 1B.

The population structure of the 153 accessions belonging to nine populations was analyzed by using STRUCTURE V2.3.4. There were two peaks for Δ*K* by Evanno’s method [55], at *K* = 2 and 5(S1 Fig). This result indicated that the 153 individuals could be divided into two clusters at K = 2, namely, Cluster I and Cluster II (Fig 1A). Cluster I contained 92 individuals, among which 19 accessions were from Pop1, two accessions were from Pop2, 18 accessions came from Pop3, 24 accessions were from Pop4, two accessions came from Pop5, 14 accessions were from Pop7, seven accessions came from Pop8, and six accessions were from Pop9. Most of individuals were from the region of south Yangtze River. Cluster II contained 61 accessions, where 44 individuals came from the north area of the Yangtze River and 17 accessions came from the south area of the Yangtze River.

There were thirteen accessions with membership probability lower than 0.60 of belonging to one subpopulation at K = 5 (Fig 1B, S2 Table). They were assigned to an ‘admixed’ group. Cluster 1 included 34 individuals, 85.29% of their members came from the south of Yangtze River. Cluster 2 (26 accessions) included accessions from Pop2, Pop6, Pop7 and Pop9. Cluster 3 contained 30 accessions, which all are members from the north of the Yangtze River. Cluster 4 (14 accessions) was only composed by the accessions from Luhu. Cluster 5 contained 33 accessions from the south of Yangtze River and 3 accessions from the north of Yangtze River.

**Fig 2:**
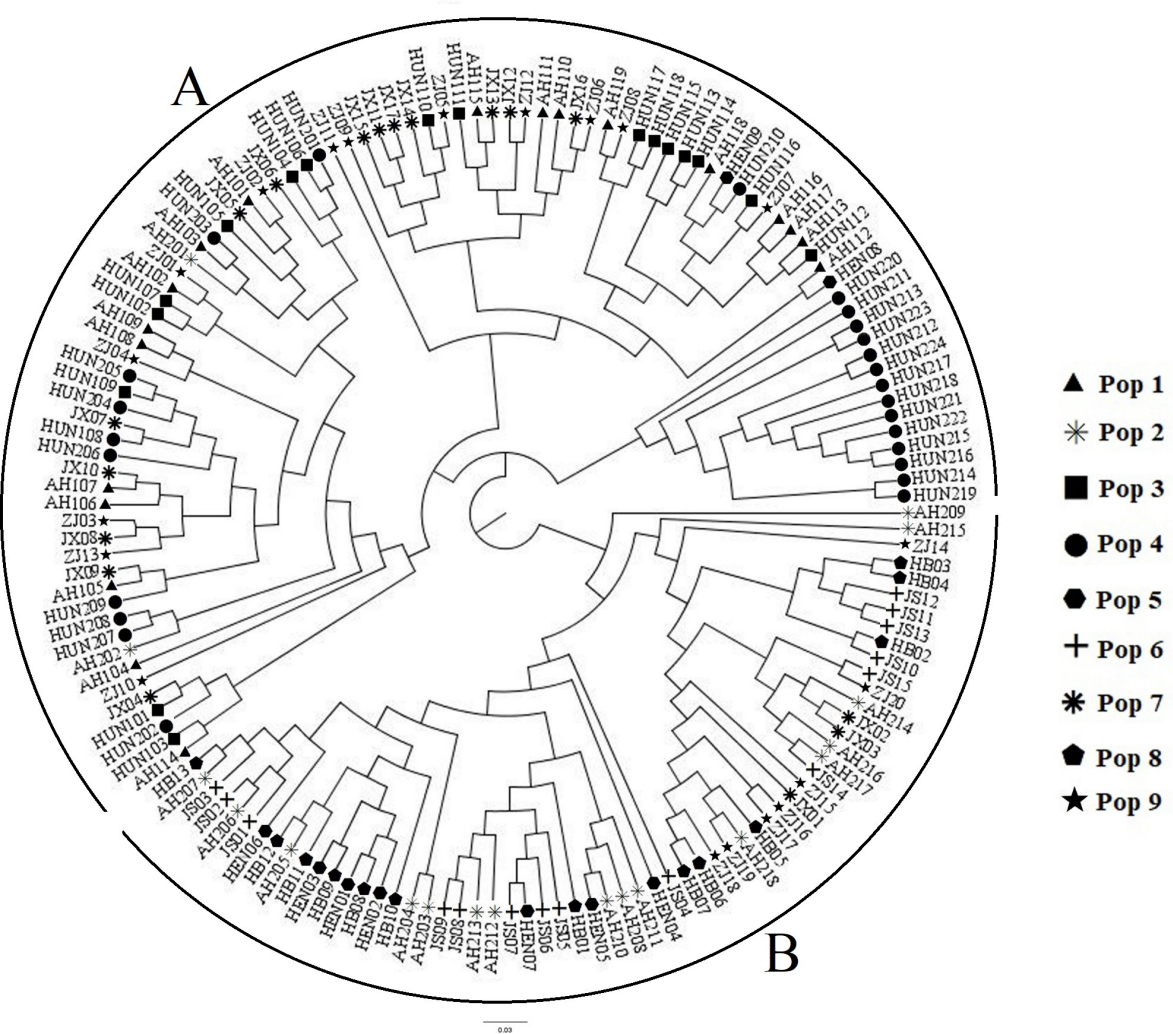
Dendrogram showing the relationship among 153 individuals of *Miscanthus lutarioriparius* based on UPGMA analysis.

A dendrogram was created using the unweighted pair group method with arithmetic mean (UPGMA) algorithm and cluster analysis, and the result is shown in Fig 2. Five clear clusters were recovered according to the genetic distance. 14 accessions of Pop4 formed a single cluster, and these accessions were all from Luhu in the Dongting Lake. The individuals of other populations were scattered among different clusters. All individuals were grouped into two main categories, as A and B. Most accessions from south of the Yangtze River merged together and formed cluster A. It contained 3 sub-clusters, including a total of 91 individuals. Cluster B contained two sub-clusters, and most of individuals were primarily collected from Hubei, He’nan, north of Anhui, and Jiangsu. There were 62 individuals in this cluster. This result was similar to the result of the STRUCTURE analysis at *K* = 5.

**Fig 3:**
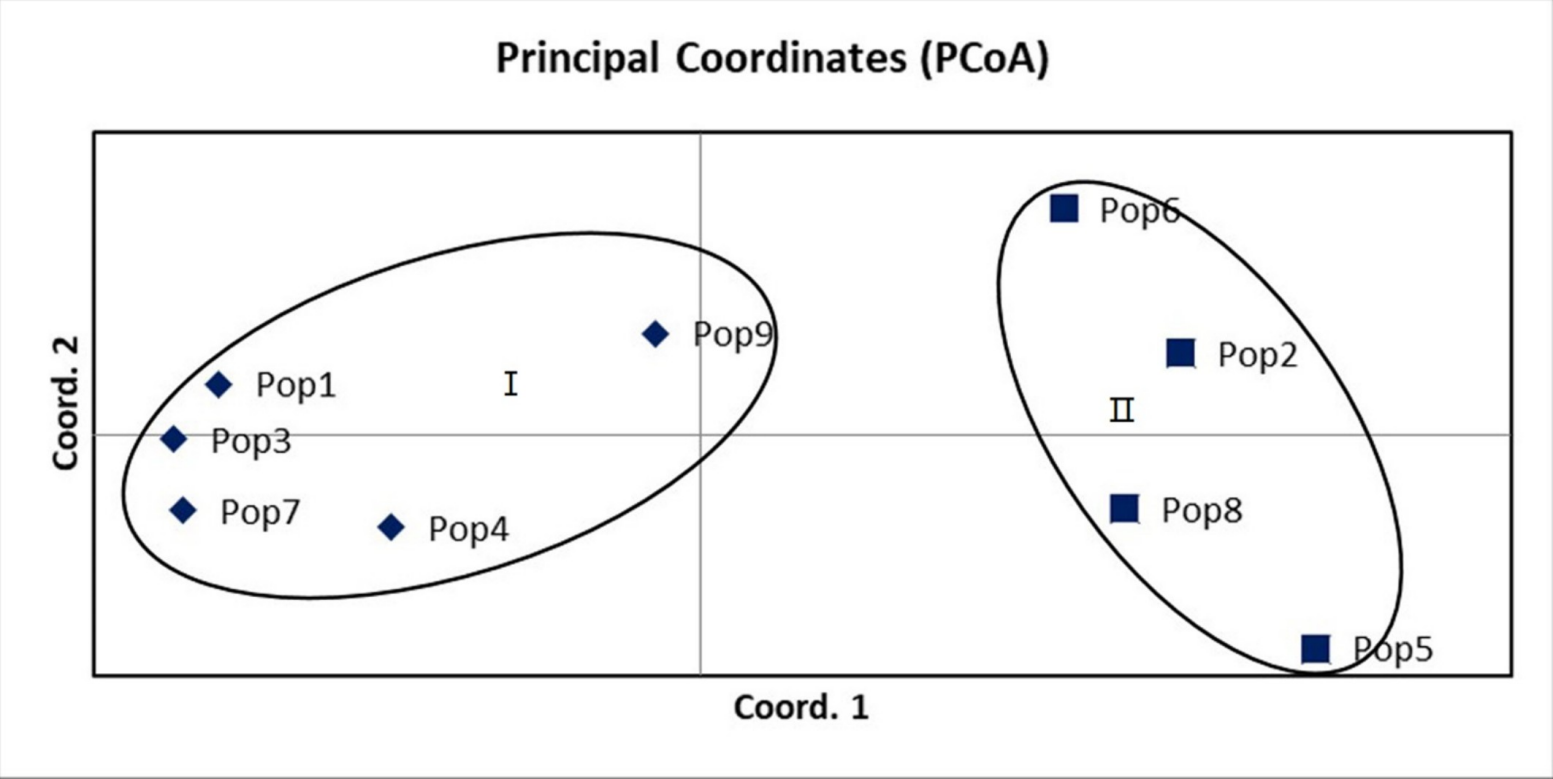
Principal coordinates analysis of nine populations of *Miscanthus lutarioriparius* based on the genetic similarity matrix derived from the combined data of SCoT markers.

The principal coordinate analysis (PCoA) for 9 populations of *M*. *lutarioriparius* revealed that these populations divided into 2 groups (Fig 3). All the populations from the south of the Yangtze River gathered into group Ⅰ, and the group Ⅱ contained 4 populations of north of the Yangtze River. Then the first principal vector accounting for 78.44% of the genetic variance, the second principle vector accounted for 10.70% and the third principle vector explaining 3.21%. The results of PCoA were the same from the other cluster analyses as shown above.

**Fig 4:**
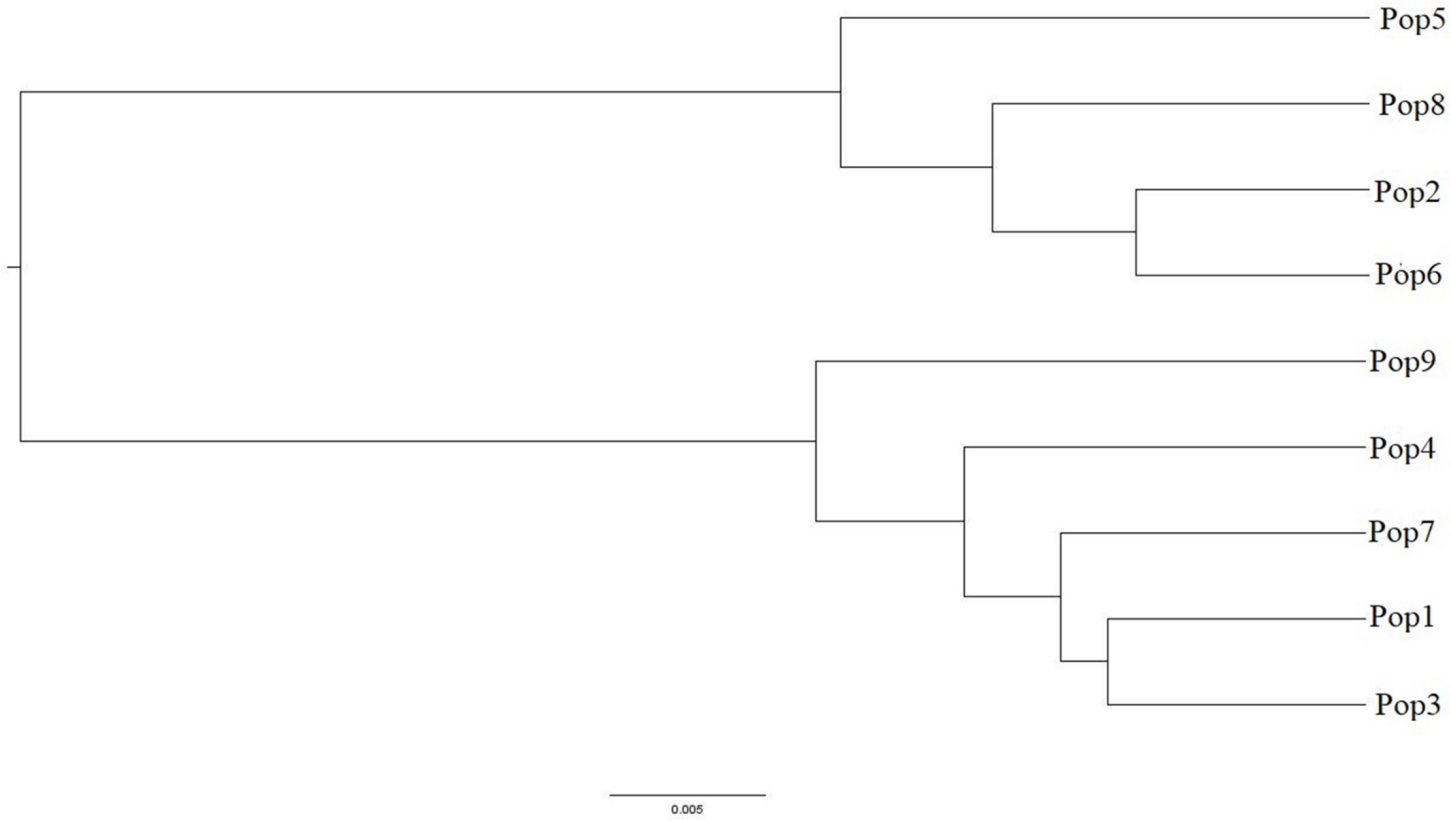
UPGMA dendrogram of nine populations of *Miscanthus lutarioriparius* based on SCoT markers.

**Fig 5:**
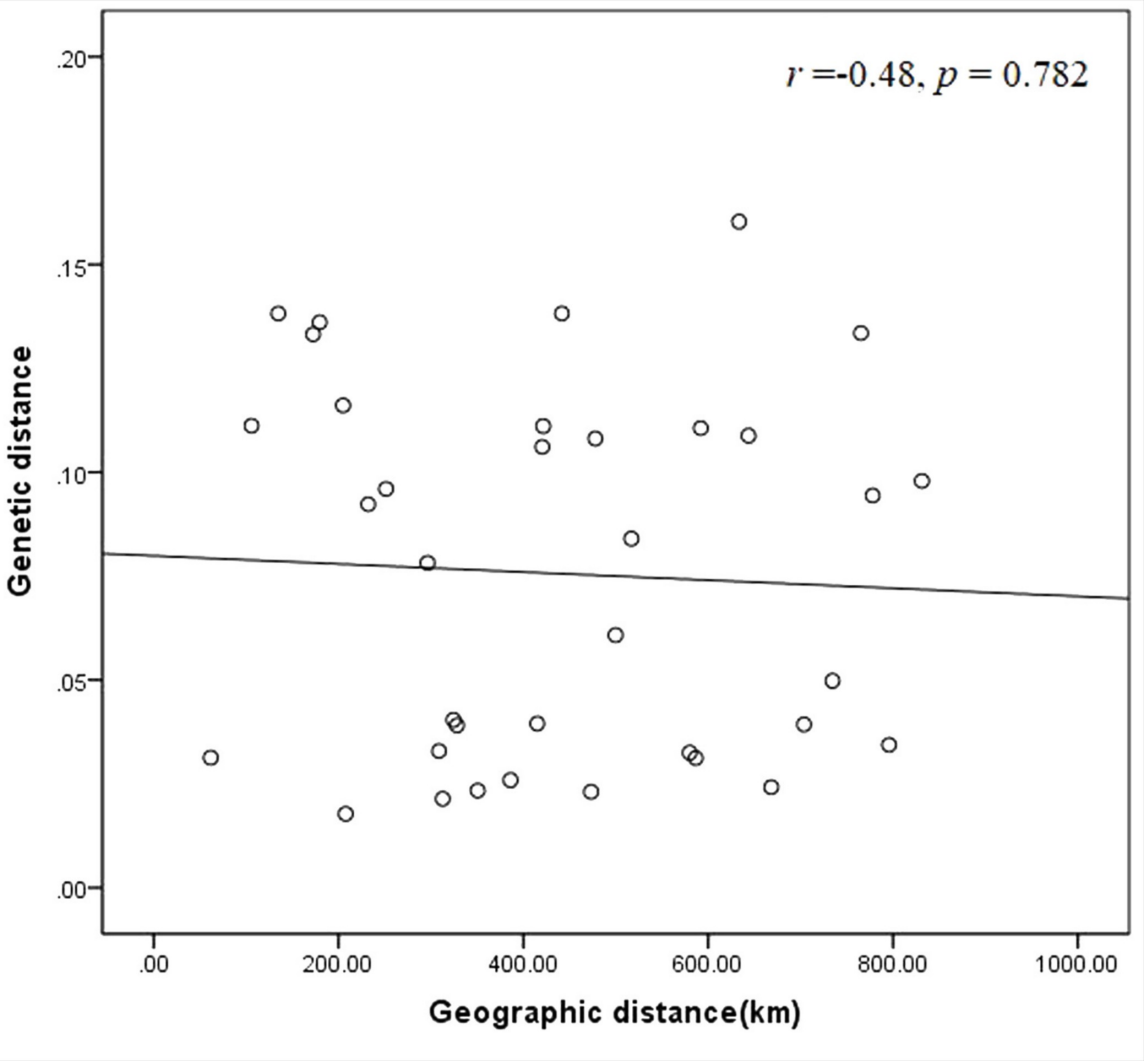
Relationship between genetic and geographic distance in the populations of *M*. *lutarioriparius*.

Genetic distance among the nine populations were calculated by the software of POWERMARKER v3.25, which could clearly reflect the genetic relationships (S3 Table). Pairwise comparisons of populations indicated relative genetic distances between populations ranging from a minimum of 0.0178 to a maximum of 0.1603 with a mean of 0.0756. The genetic distance between Pop2 from the northern of Anhui and Pop6 from Jiangsu was the closest (0.018). The distance between Pop8 from Hubei and Pop9 from Zhejiang was the farthest (0.1603). The populations of *M*. *lutarioriparius* were separated into two clusters in the UPGMA dendrogram (Fig 4). Cluster Ⅰ consisted of 4 populations, as Pop 5, Pop8, Pop2 and Pop6. All populations were from the north of the Yangtze River. Cluster Ⅱ contained Pop1, Pop3, Pop4, Pop7 and Pop9. There was no obvious correlation between genetic distance and geographic distance (r = -0.48, P = 0.782) (Fig 5).

### Genetic differentiation and gene flow

The analysis of molecular variance (AMOVA) among populations showed that 15% of the genetic differentiation (*F*_st_) was found among the populations, and the variance within populations was 85% (Table 3). The genetic variation between and within populations of *M*. *lutarioriparius* was both significant (P<0.001). The results showed that variation was more abundant within populations than among populations, and the genetic variation within populations was the main source of total variation.

**Table 3:** Result of analysis of molecular variance (AMOVA) for nine natural populations of *Miscanthus lutarioriparius*.

source of variation	d.f.	Sum of squares	Variance components	Percentage of Variance (%)	F-statistic	p-value
Among Populations	8	2483.13	11.06	15.09	*F*_*st*_ = 0.15	<0.001
Within Populations	144	8963.99	62.25	84.91		
Total	152	11447.12	73.31	100		

Results of S4 Table revealed different level of gene flow among populations. The values of gene flow (*Nm*) within populations from in the north Yangtze River area ranged from 1.32 to 24.75. The *Nm* within populations from the south of Yangtze River group ranged from 1.42 to 22.75. The results show that there was stronger connection within populations from the north area of Yangtze River, which is also true for the south group of Yangtze River. The low level of gene flow was found between populations from the north and the south of Yangtze River (ranging from 0.31 to 1.12), suggesting that the Yangtze River can server as a barrier to gene flow between populations on the both sides of the river.

## Discussion

### The universality of SCoT primers

SCoT markers are novel molecular markers that target the translation initiation site and preferentially bind to genes that are actively transcribed. These primers have been shown to exhibit relatively high levels of polymorphism [45]. It was more informative than IRAP and ISSR for the assessment of diversity of plants [60]. Here, we screened 27 SCoT primers to study the genetic diversity of *M*. *lutarioriparius* germplasm resources and detected the relationship among them. In the present investigation of *M*. *lutarioriparius*, SCoT makers displayed a high percentage of polymorphism (97.67%) and moderate PIC values of 0.26. This result indicates that SCoT markers were highly polymorphic and informative. To date, there are no studies reporting the genetic diversity of *M*. *lutarioriparius*. However, the values of PPB and PIC of SCoT markers were consistent with other molecular makers used in *M*. *sinensis* [38], *M*. *sacchariflorus* and *M*. × *giganteus* [61]. The PIC values of *M*. *lutarioriparius* were low compared with *Hemarthria* [62], *Elymus sibiricus* [63], and other plants. This might be due to different research materials and different numbers of individuals in each population. Taken together, SCoT markers can be used as an effective tool for studying *M*. *lutarioriparius*, laying the foundation for identifying *M*. *lutarioriparius* germplasm resources, genetic map construction, gene mapping, and cloning.

### Genetic diversity analysis of *M*. *lutarioriparius* germplasm

*Miscanthus lutarioriparius* is not only important as a fine raw material for papermaking, but also as a biofuel crop and ecological improvement plant. Therefore, it is important to understand the genetic diversity of *M*. *lutarioriparius* populations and the potential for genetic improvement. In this study, the mean of genetic diversity within each population was 0.32, which was similar to Chinese and U.S. *M*. *sinensis* although different markers were used in these study [23, 24, 64], and higher than previous measurements from cross-pollinated plants (*He* = 0.162) and monocotyledons (*He* = 0.181) [59, 65]. At the species level, the genetic diversity of *M*. *lutarioriparius* was similar to *Panicum virgatum* [66], *Maytenus emarginata* [67] and *Ziziphus mauritiana* [68], which were analyzed by the same markers. But it was lower than microsatellite markers [34]. This may be related to the different types of makers used.

Genetic diversity of populations is influenced by many factors, including breeding systems, population size, genetic drift, natural selection, mutation rate, and gene flow [69, 70]. First, genetic diversity at the species level is greatly related to the breeding system of *Miscanthus*, which are cross-pollinated plants that have self-incompatibility. *Miscanthus lutarioriparius* can propagate through subterraneous stems under its natural state, as well as through sexual propagation through seeds. These reproductive characteristics have vital significance for maintaining the genetic diversity in populations [71], likely resulting in higher genetic diversity. Second, *M*. *lutarioriparius* has a relatively large distribution area from 111° to 120° E and 26° to 33° N according to our field research. Generally, the larger the distribution area, the greater the genetic diversity [72]. *Miscanthus lutarioriparius* is a perennial herb, and its perennial long-life habits could provide more opportunity to accumulate mutations or special microstructure in different populations due to biotic processes. Third, since the 1950s, *M*. *lutarioriparius* has attracted attention as a paper-making material, especially in Huanan and Hubei provinces, where large-scale introduction and variety screening have taken place. Therefore, these types of perennial plants preserve variants between generations, thus increasing the genetic diversity of populations [73].

In generally, the genetic diversity of populations in downstream of river was higher than that of populations in the upper and middle reaches of a river. The genetic diversity of Pop9 was the highest, it located in the downstream of the Yangtze River. However, the populations from the middle reaches of the Yangtze River (Pop3, Pop4, Pop8 and Pop7) showed higher genetic diversity than those in the downstream Yangtze River (Pop1, Pop2 and Pop6). Pop3 and Pop4 located in the Dongting Lake area, where contains the largest, continuous distribution of this species in China. However, in Jiangsu, He’nan, and Anhui, however, the population size of *M*. *lutarioriparius* were small, with only a few individuals found, due to predatory collection and habitat destruction. Population size correlates significantly with genetic diversity [74, 75], and this was consistent with the low genetic diversity we observed in *M*. *lutarioriparius* from these areas. In the natural environment, a low seed germination rate and vegetative reproduction through the rhizomes lead to low genetic diversity within populations in small population.

### Genetic differentiation and gene flow of *Miscanthus lutarioriparius*

The genetic structures of populations are extremely important for the evolution and adaptation of a species [76]. Significant genetic differentiation was found between populations that came from the either side of Yangtze River. *Miscanthus lutarioriparius* exhibited moderate differentiation among populations. Our results were not consistent with previous studies of Yan [34]. This could be explained by our wider geographical accession collection and different molecular markers used in this study. Many factors might be responsible for moderate population differentiation, including, but not limited to pollen and seed transfer, breeding system, geographic isolation, and environment heterogeneity. In present study, the most plausible explanation for moderate genetic differentiation could be due to geographical isolation of *M*. *lutarioriparius* populations.

The results of AMOVA analysis showed that 15% of the variance was attributed to the differences among populations (P < 0.001), and 85% was attributed to the differences among individuals. The greater similarity of populations among rather than within individuals indicated that these populations were probably founded by many individuals and that there were high levels of gene flow among populations. Cross-pollinated plants often show huge variation within populations and a lower level of variation between populations [24, 25, 63]. Genetic variation of US naturalized populations of *M*. *sinensis* within populations was substantially greater than between populations [24]. *M*. *sinensis* in Southwest China showed the similar result [25]. The population genetic structures of *M*. *lutarioriparius* were similar to *M*. *sinensis*. The genetic variations in *M*. *lutarioriparius* populations was mainly derived from the genetic variation within populations.

The *N*_m_ was high within *M*. *lutarioriparius* populations from the north group and south group of Yangtze River. When *N*_m_ is higher than 1, gene flow could prevent genetic differentiation between populations caused by genetic drift [77]. Factors affecting gene flow include the dispersal modes of pollens and seeds, as well as the population size in the natural distribution. *Miscanthus lutarioriparius* is wind-pollinated, and its gene flow rate is consistent with findings that cross-pollinated plants have high gene flow [78]. *Miscanthus lutarioriparius* mainly grows along rivers and lakes, and seasonal floods can transfer seeds and subterraneous stems between different regions, further promoting genetic exchange between *M*. *lutarioriparius* populations. In addition, since the 1950s, *M*. *lutarioriparius* has been a vital raw material for paper making, and this plant was introduced widely to the area; therefore, humans have also contributed to the increased gene flow between different *M*. *lutarioriparius* populations.

Analysis indicated that gene flow was low between *M*. *lutarioriparius* populations from the north group and south group of Yangtze River. The River and mountains were usually the main reasons for limited gene communication. *M*. *lutarioriparius* was mainly distributed at elevations below 300 m (altitude) by field investigation. For example, the easternmost individuals of Pop 8 are very close to the Pop 7, but the two places were isolated by the Yangtze River, Dabie Mountain and Luoxiao Mountain. The gene flow (0.62) between the two populations was very low. Therefore, the geographic isolation could have prevented gene flow between the north group and south group of Yangtze River.

### Genetic relationships among populations of *Miscanthus lutarioriparius*

In population genetics research, sampling strategies have a strong relationship with the reliability of research results because individual localities will affect genetic diversity of a population and the population’s genetic structure [79]. Determination of a suitable population size, which can realistically reflect the population’s genetic information, is difficult because a relatively small population might originate from asexual reproduction from one or a few origins. In addition, without biotic and abiotic damage, *M*. *lutarioriparius* could theoretically enter an infinite growth cycle. Under such a scenario, it would be difficult to determine the extent of each genotype by the range of plant growth on the ground. In this research, only scattered populations were found out of the large-scale concentrated range at in some distribution areas beside the coastal area of DongTing Lake and TaiHu Lake. In these areas, the number of individuals in each population is limited and populations are separated by mountains and rivers. There was no obvious correlation between genetic distance and geographical distance. This indicated that our increased sample scale and genetic diversity at the population level does not have a significant relationship. Additionally, it demonstrated that our sample scale is reasonable and that sampled individuals are included in populations.

The genetic structure of a population is not directly correlated with its geographical distribution. According to the analysis of structure, *M*. *lutarioriparius* was divided into two groups: those distributed to the north and to the south of the Yangtze River. To further distinguish the origins of the *M*. *lutarioriparius* populations, we used PCoA and UPGMA to study the distributions of *M lutarioriparius*, resulting again in classification into two types with the Yangtze River as a natural boundary between them. Judging by certain historical records, *M*. *lutarioriparius* was considered as a member of Phragmites in the past, being introduced across China for the last 50 years as the papermaking industry has expanded. Therefore, rather than relying on pollen and seed diffusion, human activities have had a more positive influence on the distribution of *M*. *lutarioriparius*.

Here, populations with smaller geographical distances were not clustered together, while the populations on either side of the Yangtze River were clustered together. This may be a result of population isolation along the Yangtze River, causing the populations (Pop1 and Pop2) with smaller geographical distances to generate certain differences. Similar findings have been observed in other species [80].

### Protection strategies and utilization of wild *M*. *lutarioriparius* resources

*Miscanthus lutarioriparius* is endangered in some regions, this is likely not due to a loss of genetic diversity. According to our field investigation of *M*. *lutarioriparius* over many years, human activities have severely damaged the habitats of *M*. *lutarioriparius*. For example, there was large-scale *M*. *lutarioriparius* distribution in He’nan, Jiangsu, and Hubei from the 1950s to the 1970s, but now only small populations of this species are found in this area. In Hunan, after the recession of the papermaking industry, the habitat of *M*. *lutarioriparius* was used to plant poplar, leading to the decline of *M*. *lutarioriparius* populations. Therefore, *M*. *lutarioriparius* must be protected. We applied Andrew’s method to estimate the sampling size needed to protect these populations [81]. The results indicated that sampling in only one population could preserve 95% of the genetic differentiation of the species. Based on this, we recommend a protection strategy as follows: (1) preservation of the *M*. *lutarioriparius* population in the Dongting Lake area; (2) increasing the sampling of other populations with high genetic diversities (including Hubei, Jiangxi and Jiangsu), and collecting subterraneous stems to encourage reproduction during sampling.

*Miscanthus lutarioriparius* has adapted to various conditions and plays an important role in the wetland ecological environment. Simultaneously, *M*. *lutarioriparius* is considered to have potential as a second-generation cellulosic energy plant and biomaterial. For adapting to the requirements of developing energy plants in China, breeding of *M*. *lutarioriparius* germplasm suitable for growing in marginal land is necessary; therefore, understanding the genetic diversity level of *M*. *lutarioriparius* germplasm resources is the foundation for hybrid breeding. Using the results from this study, we can select distantly related materials as parents that give rise to offspring with higher genetic variation, providing better resources for selective breeding. For example, the materials in Hubei and Zhejiang populations with the greatest genetic distance could be used as parents.

## Conclusions

In conclusion, the SCoT marker system provides a highly efficient, reproducible and powerful tool for studying the genetic diversity and population structure of *M*. *lutarioriparius*. The results revealed that high genetic diversity and gene flow were detected at species level, which were attributed to its breeding system, large distribution area and anthropogenic movement of plant material. In addition, moderate genetic differentiation was found among populations, which was supported by habitat fragmentation and geographical isolation. Nine populations can be divided into two main groups by STRUCTURE analysis, PCoA and UPGMA, with the Yangtze River as a natural boundary between the two groups. All *M*. *lutarioriparius* accessions could be divided into two groups, with 92 accessions in Cluster A and 61 accessions in Cluster B. Lastly, we offered scientific measures for *M*. *lutarioriparius* protection. Thus, these results should help for selecting parents in hybridized breeding to exploiting new *Miscanthus* species and for further utilization in biomass energy and conservation.

## Supporting information

S1 Fig Result of the Bayesian assignment analysis using the Structure Harvester. (TIF)

S1 Table Sampling details of 153 *Miscanthus lutarioriparius* accessions in the study. (DOCX)

S2 Table Proportion of membership of each pre-defined population in each of the 5 clusters. (DOCX)

S3 Table Nei’s Genetic distances (below diagonal) of 9 populations of *Miscanthus lutarioriparius*. (DOCX)

S4 Table Gene flow (*Nm*) among 9 populations. (DOCX)
