# Supplementary figures and images for: Genetic diversity and population structure of Miscanthus lutarioriparius, an endemic plant of China
Source: PLoS One. 2019 Feb 1;14(2):e0211471. doi: 10.1371/journal.pone.0211471 (PMC6358086; doi:10.1371/journal.pone.0211471)

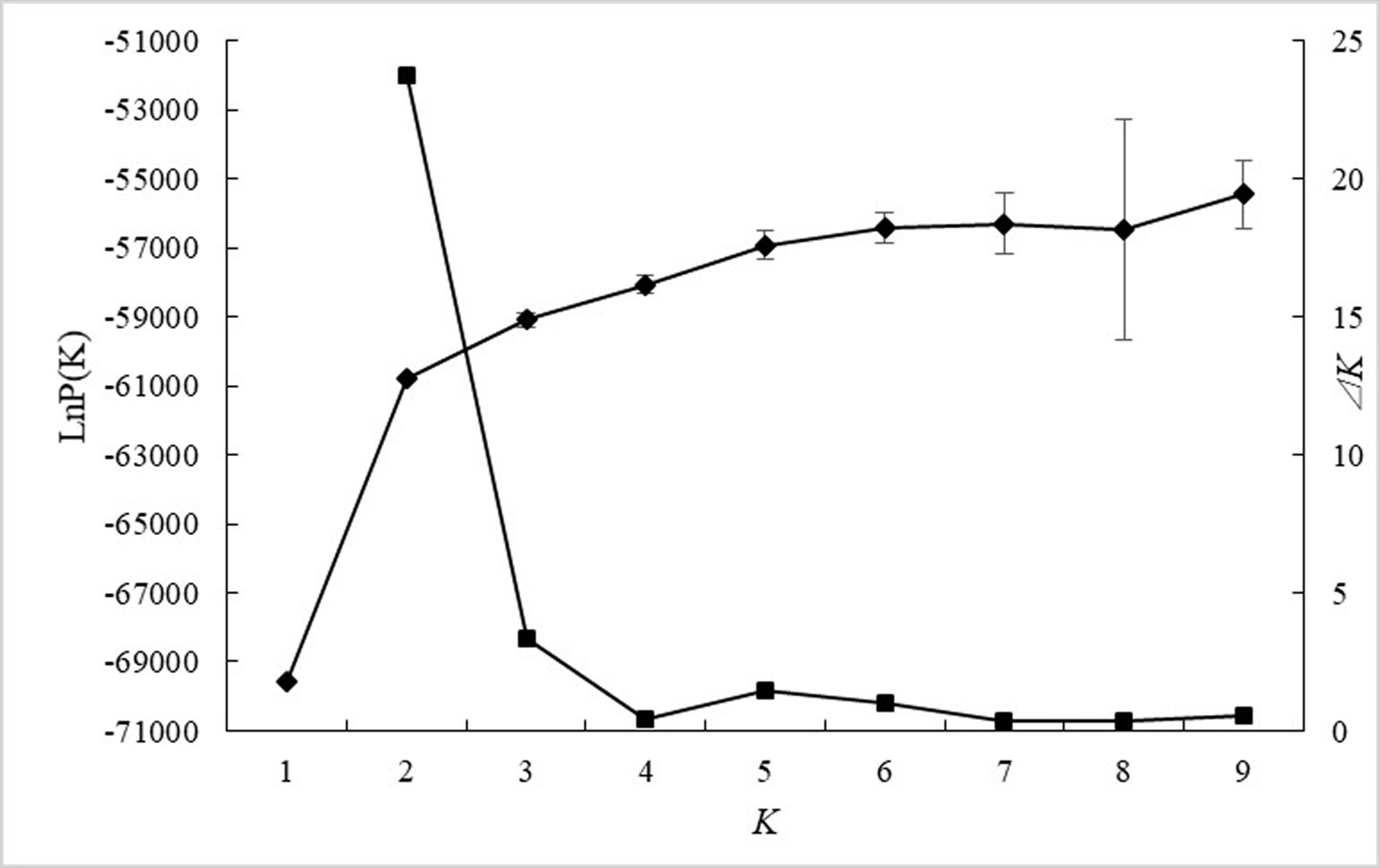

Supplement: S1 Fig — (TIF) [file pone.0211471.s001.tif]
