# Supplementary material for: Genetic diversity and population structure of Miscanthus lutarioriparius, an endemic plant of China
Source: PLoS One. 2019 Feb 1;14(2):e0211471. doi: 10.1371/journal.pone.0211471 (PMC6358086; doi:10.1371/journal.pone.0211471)
Supplement: S1 Table — (DOCX) [file pone.0211471.s002.docx]

**S1 Table Geographical parameters and the population information for *Miscanthus laturaioriprius* germplasm accessions.**

| **Code** | **Popultion** | **lantitude** | **longtitude** | **Origin** | **alltitdue** |
| --- | --- | --- | --- | --- | --- |
| AH101 | Pop 1 | 30°4'37.44"N | 116°48'35.76"E | Dongzhi, Anhui | 35 |
| AH102 | Pop 1 | 30°10'38.36"N | 116°54'13.52"E | Dongzhi, Anhui | 12 |
| AH103 | Pop 1 | 30°13'19.20"N | 116°57'41.76"E | Dongzhi, Anhui | 26 |
| AH104 | Pop 1 | 30°29'25.44"N | 117° 3'39.96"E | Dongzhi, Anhui | 13 |
| AH105 | Pop 1 | 30°36'58.59"N | 117°17'19.60"E | Chizhou, Anhui | 14 |
| AH106 | Pop 1 | 30°42'9.51"N | 117°30'36.89"E | Chizhou, Anhui | 16 |
| AH107 | Pop 1 | 30°51'41.40"N | 117°44'15.00"E | Zongyang, Anhui | 7 |
| AH108 | Pop 1 | 31°6'2.52"N | 117°59'52.08"E | Fancang, Anhui | 8 |
| AH109 | Pop 1 | 31°8'35.52"N | 118° 8'24.00"E | Fancang, Anhui | 100 |
| AH110 | Pop 1 | 31°22'46.20"N | 118°24'16.56"E | Wuhu, Anhui | 10 |
| AH111 | Pop 1 | 31°40'27.12"N | 118°31'54.84"E | Wuhu, Anhui | 22 |
| AH112 | Pop 1 | 30°6'8.28"N | 118°12'39.24"E | Huangshan, Anhui | 63 |
| AH113 | Pop 1 | 30°7'57.72"N | 118°14'12.12"E | Huangshan, Anhui | 63 |
| AH114 | Pop 1 | 30°21'14.76"N | 118°27'38.88"E | Jinde, Anhui | 77 |
| AH115 | Pop 1 | 30°32'9.96"N | 118°26'57.84"E | Jingxian, Anhui | 65 |
| AH116 | Pop 1 | 30°54'59.40"N | 118°43'16.32"E | Xuancheng, Anhui | 53 |
| AH117 | Pop 1 | 31°13'55.42"N | 118°48'36.79"E | Xuancheng, Anhui | 14 |
| AH118 | Pop 1 | 31°6'57.91"N | 118°36'55.47"E | Wuhu, Anhui | 28 |
| AH119 | Pop 1 | 31°3'31.21"N | 118°32'33.58"E | Wuhu, Anhui | 23 |
| AH201 | Pop 2 | 30°54'37.92"N | 117°34'38.64"E | Tongling, Anhui | 14 |
| AH202 | Pop 2 | 31°10'58.47"N | 117°47'12.31"E | Wuwei, Anhui | 13 |
| AH203 | Pop 2 | 31°35'50.98"N | 118°06'15.78"E | Hanshan, Anhui | 63 |
| AH204 | Pop 2 | 31°44'8.52"N | 117°19'28.83"E | Hefei, Anhui | 12 |
| AH205 | Pop 2 | 32°25'44.00"N | 116°18'28.00"E | Huoqiu, Anhui | 21 |
| AH206 | Pop 2 | 32°35'51.00"N | 116°15'50.00"E | Yinshang, Anhui | 24 |
| AH207 | Pop 2 | 32°40'58.00"N | 116°16'32.00"E | Yinshang, Anhui | 26 |
| AH208 | Pop 2 | 32°37'40.00"N | 116°46'04.00"E | Liu'An, Anhui | 63 |
| AH209 | Pop 2 | 32°32'56.00"N | 117°30'50.00"E | Dingyuan, Anhui | 84 |
| AH210 | Pop 2 | 32°45'00.00"N | 117°57'14.00"E | Mingguang, Anhui | 28 |
| AH211 | Pop 2 | 33°26'13.00"N | 117°54'36.00"E | Shouxian, Anhui | 19 |
| AH212 | Pop 2 | 31°47'58.54"N | 116°30'11.13"E | Liu'An, Anhui | 46 |
| AH213 | Pop 2 | 31°54'21.00"N | 118°02'24.00"E | Quanjiao, Anhui | 14 |
| AH214 | Pop 2 | 31°32'24.00"N | 117°00'00.00"E | Shucheng, Anhui | 12 |
| AH215 | Pop 2 | 32°13'45.85"N | 116°50'3.88"E | Shouxian, Anhui | 29 |
| AH216 | Pop 2 | 32°28'16.40"N | 116°55'1.46"E | Huainan, Anhui | 29 |
| AH217 | Pop 2 | 33°15'8.76"N | 117°49'39.32"E | Wuhe, Anhui | 17 |
| AH218 | Pop 2 | 33°4'5.49"N | 117°54'13.36"E | Wuhe, Anhui | 18 |
| HUN101 | Pop 3 | 27°47'34.89"N | 112°56'36.32"E | Xiangtan, Hunan | 50 |
| HUN102 | Pop 3 | 27°51'23.25"N | 113° 5'10.81"E | Zhuzhou, Hunan | 58 |
| HUN103 | Pop 3 | 27°55'47.74"N | 113°11'10.25"E | Zhuzhou, Hunan | 68 |
| HUN104 | Pop 3 | 27°59'38.19"N | 112°57'54.75"E | Xiangtan, Hunan | 54 |
| HUN105 | Pop 3 | 28°17'21.82"N | 112°56'22.85"E | Wangcheng, Hunan | 43 |
| HUN106 | Pop 3 | 28°26'53.56"N | 113° 9'8.06"E | Changsha, Hunan | 80 |
| HUN107 | Pop 3 | 28°24'46.99"N | 112°49'56.83"E | Wangcheng, Hunan | 35 |
| HUN108 | Pop 3 | 28°26'41.74"N | 112°56'29.46"E | Wangcheng, Hunan | 35 |
| HUN109 | Pop 3 | 28°35'23.04"N | 112°48'57.50"E | Xiangyin, Hunan | 27 |
| HUN110 | Pop 3 | 28°40'27.56"N | 112°54'38.87"E | Xiangyin, Hunan | 34 |
| HUN111 | Pop 3 | 28°44'15.92"N | 112°53'7.16"E | Xiangyin, Hunan | 34 |
| HUN112 | Pop 3 | 28°48'11.09"N | 112°52'39.66"E | Xiangyin, Hunan | 28 |
| HUN113 | Pop 3 | 28°51'18.72"N | 112°56'28.99"E | Xiangyin, Hunan | 26 |
| HUN114 | Pop 3 | 28°52'6.85"N | 113° 3'42.28"E | Miluo, Hunan | 37 |
| HUN115 | Pop 3 | 28°54'22.60"N | 112°56'5.79"E | Miluo, Hunan | 26 |
| HUN116 | Pop 3 | 28°58'7.62"N | 112°54'10.07"E | Miluo, Hunan | 25 |
| HUN117 | Pop 3 | 29°3'32.41"N | 113° 2'4.56"E | Yueyang, Hunan | 38 |
| HUN118 | Pop 3 | 29°15'59.90"N | 113° 5'31.27"E | Yueyang, Hunan | 57 |
| HUN201 | Pop 4 | 30°0'36.77"N | 110°42'23.74"E | Shimen, Hunan | 79 |
| HUN202 | Pop 4 | 29°34'18.95"N | 112°15'43.71"E | Anxiang, Hunan | 32 |
| HUN203 | Pop 4 | 29°28'32.87"N | 112°16'15.86"E | Anxiang, Hunan | 31 |
| HUN204 | Pop 4 | 29°31'4.42"N | 112° 4'20.55"E | Anxiang, Hunan | 32 |
| HUN205 | Pop 4 | 29°30'50.94"N | 112°37'10.34"E | Huarong, Hunan | 38 |
| HUN206 | Pop 4 | 29°31'18.47"N | 112°39'17.62"E | Huarong, Hunan | 38 |
| HUN207 | Pop 4 | 29°28'3.78"N | 112°48'33.56"E | Yueyang, Hunan | 26 |
| HUN208 | Pop 4 | 29°23'15.88"N | 112°49'30.14"E | Huarong,Hunan | 28 |
| HUN209 | Pop 4 | 29°24'28.92"N | 112°53'3.51"E | Yueyang, Hunan | 26 |
| HUN210 | Pop 4 | 29°3'39.63"N | 112°22'11.47"E | Yuanjiang, Hunan | 30 |
| HUN211 | Pop 4 | 29°4'13.67"N | 112°27'48.64"E | Yuanjiang, Hunan | 28 |
| HUN212 | Pop 4 | 29°2'59.80"N | 112°34'34.01"E | Yuanjiang, Hunan | 28 |
| HUN213 | Pop 4 | 28°59'35.55"N | 112°41'47.79"E | Yuanjiang, Hunan | 28 |
| HUN214 | Pop 4 | 28°57'53.28"N | 112°50'12.34"E | Yuanjiang, Hunan | 25 |
| HUN215 | Pop 4 | 28°57'48.54"N | 112°50'57.64"E | Yuanjiang, Hunan | 25 |
| HUN216 | Pop 4 | 28°58'31.51"N | 112°51'8.64"E | Yuanjiang, Hunan | 25 |
| HUN217 | Pop 4 | 28°56'25.37"N | 112°50'5.85"E | Yuanjiang, Hunan | 25 |
| HUN218 | Pop 4 | 28°57'18.58"N | 112°51'29.19"E | Xiangyin, Hunan | 25 |
| HUN219 | Pop 4 | 28°54'0.64"N | 112°22'10.76"E | Yuanjiang, Hunan | 31 |
| HUN220 | Pop 4 | 28°52'5.46"N | 112°24'21.51"E | Yuanjiang, Hunan | 31 |
| HUN221 | Pop 4 | 28°48'33.12"N | 112°33'44.62"E | Yuanjiang, Hunan | 29 |
| HUN222 | Pop 4 | 28°48'9.83"N | 112°34'18.92"E | Yiyang, Hunan | 29 |
| HUN223 | Pop 4 | 28°46'29.39"N | 112°39'45.31"E | Xiangyin, Hunan | 29 |
| HUN224 | Pop 4 | 28°46'9.78"N | 112°45'34.77"E | Xiangyin, Hunan | 26 |
| HEN01 | Pop 5 | 33°13'29.41"N | 112°31'56.02"E | Nanyang, Henan | 164 |
| HEN02 | Pop 5 | 32°54'49.18"N | 113°13'7.16"E | Miyang, Henan | 146 |
| HEN03 | Pop 5 | 32°54'6.43"N | 111°56'49.53"E | Dengzhou, Henan | 146 |
| HEN04 | Pop 5 | 32°44'8.77"N | 112°16'26.83"E | Dengzhou, Henan | 99 |
| HEN05 | Pop 5 | 32°27'45.89"N | 112°33'11.68"E | Nanyang, Henan | 85 |
| HEN06 | Pop 5 | 31°54'47.96"N | 114°28'4.48"E | Luoshan, Henan | 87 |
| HEN07 | Pop 5 | 32°25'5.42"N | 115°29'6.95"E | Huaibin, Henan | 31 |
| HEN08 | Pop 5 | 32°19'29.83"N | 115° 7'59.36"E | Huangcuan, Henan | 37 |
| HEN09 | Pop 5 | 32°15'15.05"N | 115°22'25.81"E | Huaibin, Henan | 36 |
| JS01 | Pop 6 | 33°34'01.00"N | 118°23'52.00"E | Sihong, Jiangsu | 15 |
| JS02 | Pop 6 | 33°56'06.00"N | 118°43'12.00"E | Sihong, Jiangsu | 9 |
| JS03 | Pop 6 | 32°30'00.00"N | 120°13'59.88"E | Jiangyan, Jiangsu | 5 |
| JS04 | Pop 6 | 32°25'22.00"N | 118°55'08.00"E | Nanjing, Jiangsu | 27 |
| JS05 | Pop 6 | 33°47'60.00"N | 118°56'06.00"E | Huaiyin, Jiangsu | 9 |
| JS06 | Pop 6 | 33°19'26.00"N | 120°24'00.00"E | Yancheng, Jiangsu | 1 |
| JS07 | Pop 6 | 33°49'30.00"N | 119°07'37.00"E | Huai'An, Jiangsu | 8 |
| JS08 | Pop 6 | 32°41'24.00"N | 119°17'60.00"E | Gaoyou, Jiangsu | 6 |
| JS09 | Pop 6 | 32°47'60.00"N | 119°37'48.00"E | Gaoyou, Jiangsu | 1 |
| JS10 | Pop 6 | 33°58'44.00"N | 119°44'56.00"E | Binhai, Jiangsu | 1 |
| JS11 | Pop 6 | 32°49'33.00"N | 119°45'46.00"E | Gaoyou, Jiangsu | 1 |
| JS12 | Pop 6 | 32°59'17.16"N | 120°48'20.16"E | Nantong, Jiangsu | 3 |
| JS13 | Pop 6 | 32°29'17.16"N | 120°48'20.16"E | Dangfeng, Jiangsu | 3 |
| JS14 | Pop 6 | 32°27'23.76"N | 119°55'22.80"E | Taizhou, Jiangsu | 5 |
| JS15 | Pop 6 | 32°59'22.92"N | 119°55'23.88"E | Taizhou, Jiangsu | 0 |
| JX01 | Pop 7 | 29°0'6.84"N | 114°13'31.44"E | Xiushui, Jiangxi | 147 |
| JX02 | Pop 7 | 29°1'55.92"N | 114°24'53.64"E | Xiushui, Jiangxi | 138 |
| JX03 | Pop 7 | 29°12'25.56"N | 115°23'58.92"E | Jiujiang, Jiangxi | 20 |
| JX04 | Pop 7 | 29°02'34.80"N | 115°48'18.00"E | Jiujiang, Jiangxi | 20 |
| JX05 | Pop 7 | 28°47'13.13"N | 115°52'15.90"E | Nancang, Jiangxi | 37 |
| JX06 | Pop 7 | 28°31'41.18"N | 115°51'55.04"E | Nancang, Jiangxi | 20 |
| JX07 | Pop 7 | 28°29'59.97"N | 115°59'57.53"E | Nancang, Jiangxi | 23 |
| JX08 | Pop 7 | 28°30'32.02"N | 116°02'1.42"E | Nancang, Jiangxi | 20 |
| JX09 | Pop 7 | 28°37'34.68"N | 116°17'30.12"E | Jinxian, Jiangxi | 17 |
| JX10 | Pop 7 | 28°44'17.16"N | 116°30'31.32"E | Yugan, Jiangxi | 21 |
| JX11 | Pop 7 | 28°55'35.76"N | 116°45'52.20"E | Poyang, Jiangxi | 15 |
| JX12 | Pop 7 | 29°24'26.00"N | 116°14'15.47"E | Ducang, Jiangxi | 20 |
| JX13 | Pop 7 | 29°27'40.72"N | 115°36'59.73"E | De'An, Jiangxi | 35 |
| JX14 | Pop 7 | 29°38'1.23"N | 115°50'21.64"E | Jiujiang, Jiangxi | 37 |
| JX15 | Pop 7 | 29°38'52.44"N | 116°05'4.92"E | Jiujiang, Jiangxi | 25 |
| JX16 | Pop 7 | 29°44'28.68"N | 116°12'40.68"E | Jiujiang, Jiangxi | 29 |
| JX17 | Pop 7 | 29°47'59.28"N | 116°22'37.56"E | Hukou, Jiangxi | 28 |
| HB01 | Pop 8 | 29°48'6.36"N | 116°02'11.68"E | Huanggang, Hubei | 56 |
| HB02 | Pop 8 | 30°13'59.74"N | 115°06'23.94"E | Huanggang, Hubei | 56 |
| HB03 | Pop 8 | 30°31'28.72"N | 114°13'36.25"E | Wuhan,Hubei | 134 |
| HB04 | Pop 8 | 30°10'2.23"N | 113°40'26.24"E | Xiantao, Hubei | 134 |
| HB05 | Pop 8 | 29°48'40.40"N | 112°51'4.83"E | Jianli, Hubei | 14 |
| HB06 | Pop 8 | 30°24'44.48"N | 112°50'54.67"E | Qianjiang, Hubei | 86 |
| HB07 | Pop 8 | 30°19'0.94"N | 112°28'25.18"E | Jingmen, Hubei | 26 |
| HB08 | Pop 8 | 30°15'6.67"N | 111°32'52.43"E | Yicheng, Hubei | 23 |
| HB09 | Pop 8 | 31°12'25.69"N | 112°15'31.97"E | Jingmen, Hubei | 30 |
| HB10 | Pop 8 | 31°43'58.32"N | 112°16'20.28"E | Yicheng, Hubei | 32 |
| HB11 | Pop 8 | 31°51'17.08"N | 112°23'44.97"E | Xiangyang, Hubei | 27 |
| HB12 | Pop 8 | 31°54'15.60"N | 112°21'24.63"E | Xiangyang, Hubei | 61 |
| HB13 | Pop 8 | 32°9'12.61"N | 112°08'1.01"E | Xiangyang, Hubei | 76 |
| ZJ01 | Pop 9 | 30°24'35.11"N | 120°36'3.14"E | Haining, Zhejiang | 7 |
| ZJ02 | Pop 9 | 31°01'48.00"N | 120°00'00.00"E | Changxing, Zhejiang | 3 |
| ZJ03 | Pop 9 | 30°53'60.00"N | 120°13'12.00"E | Huzhou, Zhejiang | 3 |
| ZJ04 | Pop 9 | 30°49'11.00"N | 120°14'23.00"E | Huzhou, Zhejiang | 4 |
| ZJ05 | Pop 9 | 30°55'48.00"N | 120°14'24.00"E | Huzhou, Zhejiang | 2 |
| ZJ06 | Pop 9 | 30°46'48.00"N | 120°25'12.00"E | Huzhou, Zhejiang | 5 |
| ZJ07 | Pop 9 | 30°09'03.00"N | 120°58'04.00"E | Yuyao, Zhejiang | 11 |
| ZJ08 | Pop 9 | 32°10'9.83"N | 119°20'23.10"E | Zhengjiang, Jiangsu | 6 |
| ZJ09 | Pop 9 | 31°24'50.00"N | 120°14'38.00"E | Wuxi, Jiangsu | 4 |
| ZJ10 | Pop 9 | 31°11'19.56"N | 119°53'52.14"E | Yixing, Jiangsu | 4 |
| ZJ11 | Pop 9 | 31°15'36.00"N | 120°23'24.00"E | Suzhou, Jiangsu | 21 |
| ZJ12 | Pop 9 | 31°23'60.00"N | 120°24'00.00"E | Suzhou, Jiangsu | 4 |
| ZJ13 | Pop 9 | 31°33'19.89"N | 119°40'10.13"E | Yixing, Jiangsu | 5 |
| ZJ14 | Pop 9 | 30°58'48.00"N | 120°25'48.00"E | Suzhou, Jiangsu | 4 |
| ZJ15 | Pop 9 | 31°08'09.00"N | 120°26'20.00"E | Suzhou, Jiangsu | 2 |
| ZJ16 | Pop 9 | 31°00'00.00"N | 120°28'12.00"E | Suzhou, Jiangsu | 4 |
| ZJ17 | Pop 9 | 31°07'12.00"N | 120°30'36.00"E | Suzhou, Jiangsu | 2 |
| ZJ18 | Pop 9 | 31°40'48.00"N | 120°31'12.00"E | Wuxi, Jiangsu | 5 |
| ZJ19 | Pop 9 | 31°20'24.00"N | 119°51'36.00"E | Yixing, Jiangsu | 4 |
| ZJ20 | Pop 9 | 31°21'00.00"N | 119°46'48.00"E | Yixing, Jiangsu | 8 |
