# Supplementary material for: Genetic diversity and population structure of Miscanthus lutarioriparius, an endemic plant of China
Source: PLoS One. 2019 Feb 1;14(2):e0211471. doi: 10.1371/journal.pone.0211471 (PMC6358086; doi:10.1371/journal.pone.0211471)
Supplement: S2 Table — (DOCX) [file pone.0211471.s003.docx]

**S2 Table Proportion of membership of each pre-defined population in each of the 5 clusters**

| **No.** | **Code** | **(%Miss)** | **Pop** | **Cluster 1** | **Cluster 2** | **Cluster 3** | **Cluster 4** | **Cluster 5** |
| --- | --- | --- | --- | --- | --- | --- | --- | --- |
| 1 | AH101 | 0 | 1 | 0.01 | 0.00 | 0.00 | 0.00 | 0.99 |
| 2 | AH102 | 0 | 1 | 0.01 | 0.01 | 0.02 | 0.04 | 0.92 |
| 3 | AH103 | 0 | 1 | 0.02 | 0.00 | 0.00 | 0.00 | 0.97 |
| 4 | AH104 | 0 | 1 | 0.23 | 0.01 | 0.01 | 0.09 | 0.66 |
| 5 | AH105 | 0 | 1 | 0.00 | 0.00 | 0.00 | 0.00 | 0.99 |
| 6 | AH106 | 0 | 1 | 0.04 | 0.00 | 0.00 | 0.01 | 0.95 |
| 7 | AH107 | 0 | 1 | 0.02 | 0.00 | 0.00 | 0.01 | 0.97 |
| 8 | AH108 | 0 | 1 | 0.52 | 0.00 | 0.01 | 0.02 | 0.45 |
| 9 | AH109 | 0 | 1 | 0.10 | 0.00 | 0.00 | 0.20 | 0.70 |
| 10 | AH110 | 0 | 1 | 0.60 | 0.00 | 0.00 | 0.01 | 0.39 |
| 11 | AH111 | 0 | 1 | 0.99 | 0.00 | 0.00 | 0.00 | 0.01 |
| 12 | AH112 | 0 | 1 | 0.76 | 0.09 | 0.00 | 0.00 | 0.15 |
| 13 | AH113 | 0 | 1 | 0.98 | 0.00 | 0.00 | 0.01 | 0.01 |
| 14 | AH114 | 0 | 1 | 0.95 | 0.00 | 0.00 | 0.02 | 0.03 |
| 15 | AH115 | 0 | 1 | 0.90 | 0.00 | 0.00 | 0.00 | 0.09 |
| 16 | AH116 | 0 | 1 | 0.95 | 0.00 | 0.00 | 0.04 | 0.01 |
| 17 | AH117 | 0 | 1 | 0.92 | 0.00 | 0.00 | 0.05 | 0.02 |
| 18 | AH118 | 0 | 1 | 0.56 | 0.00 | 0.00 | 0.27 | 0.17 |
| 19 | AH119 | 0 | 1 | 0.97 | 0.00 | 0.00 | 0.00 | 0.02 |
| 20 | AH201 | 0 | 2 | 0.14 | 0.00 | 0.00 | 0.00 | 0.85 |
| 21 | AH202 | 0 | 2 | 0.03 | 0.01 | 0.01 | 0.19 | 0.77 |
| 22 | AH203 | 0 | 2 | 0.00 | 0.23 | 0.77 | 0.00 | 0.00 |
| 23 | AH204 | 0 | 2 | 0.01 | 0.16 | 0.81 | 0.01 | 0.01 |
| 24 | AH205 | 0 | 2 | 0.00 | 0.01 | 0.98 | 0.00 | 0.01 |
| 25 | AH206 | 0 | 2 | 0.01 | 0.00 | 0.97 | 0.00 | 0.01 |
| 26 | AH207 | 0 | 2 | 0.00 | 0.00 | 1.00 | 0.00 | 0.00 |
| 27 | AH208 | 0 | 2 | 0.01 | 0.13 | 0.85 | 0.01 | 0.00 |
| 28 | AH209 | 0 | 2 | 0.00 | 0.15 | 0.64 | 0.20 | 0.00 |
| 29 | AH210 | 0 | 2 | 0.00 | 0.00 | 0.97 | 0.02 | 0.00 |
| 30 | AH211 | 0 | 2 | 0.00 | 0.19 | 0.81 | 0.00 | 0.00 |
| 31 | AH212 | 0 | 2 | 0.00 | 0.51 | 0.48 | 0.00 | 0.00 |
| 32 | AH213 | 0 | 2 | 0.00 | 0.60 | 0.40 | 0.00 | 0.00 |
| 33 | AH214 | 0 | 2 | 0.00 | 0.83 | 0.17 | 0.00 | 0.00 |
| 34 | AH215 | 0 | 2 | 0.00 | 0.86 | 0.09 | 0.03 | 0.02 |
| 35 | AH216 | 0 | 2 | 0.01 | 0.93 | 0.00 | 0.06 | 0.00 |
| 36 | AH217 | 0 | 2 | 0.00 | 0.84 | 0.15 | 0.00 | 0.00 |
| 37 | AH218 | 0 | 2 | 0.00 | 1.00 | 0.00 | 0.00 | 0.00 |
| 38 | HUN101 | 0 | 3 | 0.45 | 0.00 | 0.00 | 0.01 | 0.54 |
| 39 | HUN102 | 0 | 3 | 0.06 | 0.00 | 0.00 | 0.01 | 0.93 |
| 40 | HUN103 | 0 | 3 | 0.43 | 0.00 | 0.01 | 0.02 | 0.54 |
| 41 | HUN104 | 0 | 3 | 0.04 | 0.00 | 0.00 | 0.00 | 0.96 |
| 42 | HUN105 | 0 | 3 | 0.07 | 0.01 | 0.00 | 0.01 | 0.92 |
| 43 | HUN106 | 0 | 3 | 0.01 | 0.00 | 0.00 | 0.00 | 0.99 |
| 44 | HUN107 | 0 | 3 | 0.01 | 0.04 | 0.01 | 0.00 | 0.94 |
| 45 | HUN108 | 0 | 3 | 0.00 | 0.00 | 0.00 | 0.00 | 0.99 |
| 46 | HUN109 | 0 | 3 | 0.00 | 0.00 | 0.00 | 0.00 | 1.00 |
| 47 | HUN110 | 0 | 3 | 0.81 | 0.02 | 0.00 | 0.00 | 0.17 |
| 48 | HUN111 | 0 | 3 | 0.88 | 0.00 | 0.00 | 0.00 | 0.12 |
| 49 | HUN112 | 0 | 3 | 0.65 | 0.00 | 0.00 | 0.35 | 0.00 |
| 50 | HUN113 | 0 | 3 | 0.69 | 0.00 | 0.00 | 0.31 | 0.00 |
| 51 | HUN114 | 0 | 3 | 0.65 | 0.05 | 0.01 | 0.29 | 0.00 |
| 52 | HUN115 | 0 | 3 | 0.64 | 0.00 | 0.01 | 0.29 | 0.06 |
| 53 | HUN116 | 0 | 3 | 0.70 | 0.00 | 0.00 | 0.30 | 0.00 |
| 54 | HUN117 | 0 | 3 | 0.69 | 0.00 | 0.00 | 0.12 | 0.19 |
| 55 | HUN118 | 0 | 3 | 0.85 | 0.00 | 0.00 | 0.00 | 0.14 |
| 56 | HUN201 | 0 | 4 | 0.01 | 0.00 | 0.00 | 0.00 | 0.99 |
| 57 | HUN202 | 0 | 4 | 0.38 | 0.00 | 0.00 | 0.17 | 0.45 |
| 58 | HUN203 | 0 | 4 | 0.32 | 0.00 | 0.00 | 0.03 | 0.65 |
| 59 | HUN204 | 0 | 4 | 0.05 | 0.01 | 0.01 | 0.00 | 0.93 |
| 60 | HUN205 | 0 | 4 | 0.00 | 0.00 | 0.00 | 0.00 | 0.99 |
| 61 | HUN206 | 0 | 4 | 0.13 | 0.00 | 0.00 | 0.00 | 0.87 |
| 62 | HUN207 | 0 | 4 | 0.23 | 0.00 | 0.00 | 0.00 | 0.77 |
| 63 | HUN208 | 0 | 4 | 0.59 | 0.01 | 0.00 | 0.03 | 0.38 |
| 64 | HUN209 | 0 | 4 | 0.17 | 0.00 | 0.00 | 0.00 | 0.82 |
| 65 | HUN210 | 0 | 4 | 0.69 | 0.00 | 0.00 | 0.31 | 0.00 |
| 66 | HUN211 | 0 | 4 | 0.25 | 0.01 | 0.01 | 0.73 | 0.00 |
| 67 | HUN212 | 0 | 4 | 0.01 | 0.00 | 0.01 | 0.98 | 0.01 |
| 68 | HUN213 | 0 | 4 | 0.01 | 0.00 | 0.00 | 0.78 | 0.20 |
| 69 | HUN214 | 0 | 4 | 0.00 | 0.01 | 0.01 | 0.80 | 0.18 |
| 70 | HUN215 | 0 | 4 | 0.00 | 0.00 | 0.00 | 0.99 | 0.00 |
| 71 | HUN216 | 0 | 4 | 0.00 | 0.00 | 0.00 | 1.00 | 0.00 |
| 72 | HUN217 | 0 | 4 | 0.01 | 0.00 | 0.00 | 0.98 | 0.00 |
| 73 | HUN218 | 0 | 4 | 0.03 | 0.00 | 0.00 | 0.92 | 0.05 |
| 74 | HUN219 | 0 | 4 | 0.02 | 0.01 | 0.00 | 0.96 | 0.01 |
| 75 | HUN220 | 0 | 4 | 0.01 | 0.01 | 0.01 | 0.71 | 0.28 |
| 76 | HUN221 | 0 | 4 | 0.08 | 0.00 | 0.00 | 0.91 | 0.00 |
| 77 | HUN222 | 0 | 4 | 0.02 | 0.00 | 0.00 | 0.89 | 0.09 |
| 78 | HUN223 | 0 | 4 | 0.05 | 0.00 | 0.00 | 0.72 | 0.23 |
| 79 | HUN224 | 0 | 4 | 0.01 | 0.00 | 0.00 | 0.98 | 0.00 |
| 80 | HEN01 | 0 | 5 | 0.00 | 0.03 | 0.97 | 0.00 | 0.00 |
| 81 | HEN02 | 0 | 5 | 0.00 | 0.00 | 0.99 | 0.00 | 0.00 |
| 82 | HEN03 | 0 | 5 | 0.00 | 0.00 | 1.00 | 0.00 | 0.00 |
| 83 | HEN04 | 0 | 5 | 0.03 | 0.32 | 0.65 | 0.00 | 0.00 |
| 84 | HEN05 | 0 | 5 | 0.00 | 0.00 | 1.00 | 0.00 | 0.00 |
| 85 | HEN06 | 0 | 5 | 0.00 | 0.07 | 0.91 | 0.01 | 0.00 |
| 86 | HEN07 | 0 | 5 | 0.00 | 0.07 | 0.93 | 0.00 | 0.00 |
| 87 | HEN08 | 0 | 5 | 0.88 | 0.00 | 0.00 | 0.12 | 0.00 |
| 88 | HEN09 | 0 | 5 | 0.99 | 0.00 | 0.00 | 0.01 | 0.00 |
| 89 | JS01 | 0 | 6 | 0.00 | 0.06 | 0.94 | 0.00 | 0.00 |
| 90 | JS02 | 0 | 6 | 0.00 | 0.03 | 0.97 | 0.00 | 0.00 |
| 91 | JS03 | 0 | 6 | 0.00 | 0.00 | 0.99 | 0.00 | 0.00 |
| 92 | JS04 | 0 | 6 | 0.00 | 0.22 | 0.68 | 0.10 | 0.00 |
| 93 | JS05 | 0 | 6 | 0.00 | 0.04 | 0.95 | 0.00 | 0.00 |
| 94 | JS06 | 0 | 6 | 0.00 | 0.27 | 0.73 | 0.00 | 0.00 |
| 95 | JS07 | 0 | 6 | 0.00 | 0.00 | 1.00 | 0.00 | 0.00 |
| 96 | JS08 | 0 | 6 | 0.00 | 0.32 | 0.68 | 0.00 | 0.00 |
| 97 | JS09 | 0 | 6 | 0.00 | 0.42 | 0.57 | 0.00 | 0.00 |
| 98 | JS10 | 0 | 6 | 0.00 | 0.45 | 0.55 | 0.00 | 0.00 |
| 99 | JS11 | 0 | 6 | 0.00 | 0.83 | 0.17 | 0.00 | 0.00 |
| 100 | JS12 | 0 | 6 | 0.00 | 0.89 | 0.11 | 0.00 | 0.00 |
| 101 | JS13 | 0 | 6 | 0.00 | 0.77 | 0.23 | 0.00 | 0.00 |
| 102 | JS14 | 0 | 6 | 0.00 | 0.98 | 0.01 | 0.00 | 0.01 |
| 103 | JS15 | 0 | 6 | 0.00 | 0.78 | 0.21 | 0.00 | 0.00 |
| 104 | JX01 | 0 | 7 | 0.00 | 0.99 | 0.00 | 0.00 | 0.00 |
| 105 | JX02 | 0 | 7 | 0.00 | 0.99 | 0.00 | 0.00 | 0.00 |
| 106 | JX03 | 0 | 7 | 0.00 | 0.98 | 0.02 | 0.00 | 0.00 |
| 107 | JX04 | 0 | 7 | 0.39 | 0.03 | 0.04 | 0.12 | 0.41 |
| 108 | JX05 | 0 | 7 | 0.25 | 0.00 | 0.00 | 0.00 | 0.75 |
| 109 | JX06 | 0 | 7 | 0.00 | 0.00 | 0.00 | 0.03 | 0.96 |
| 110 | JX07 | 0 | 7 | 0.00 | 0.00 | 0.00 | 0.00 | 0.99 |
| 111 | JX08 | 0 | 7 | 0.00 | 0.00 | 0.00 | 0.00 | 0.99 |
| 112 | JX09 | 0 | 7 | 0.00 | 0.00 | 0.00 | 0.00 | 0.99 |
| 113 | JX10 | 0 | 7 | 0.00 | 0.00 | 0.00 | 0.01 | 0.99 |
| 114 | JX11 | 0 | 7 | 0.87 | 0.00 | 0.00 | 0.12 | 0.00 |
| 115 | JX12 | 0 | 7 | 0.76 | 0.00 | 0.00 | 0.00 | 0.24 |
| 116 | JX13 | 0 | 7 | 0.82 | 0.00 | 0.00 | 0.00 | 0.18 |
| 117 | JX14 | 0 | 7 | 0.84 | 0.00 | 0.00 | 0.01 | 0.15 |
| 118 | JX15 | 0 | 7 | 0.95 | 0.00 | 0.00 | 0.00 | 0.04 |
| 119 | JX16 | 0 | 7 | 0.98 | 0.00 | 0.00 | 0.02 | 0.00 |
| 120 | JX17 | 0 | 7 | 0.93 | 0.01 | 0.04 | 0.01 | 0.01 |
| 121 | HB01 | 0 | 8 | 0.00 | 0.00 | 0.00 | 0.00 | 0.99 |
| 122 | HB02 | 0 | 8 | 0.01 | 0.00 | 0.00 | 0.00 | 0.99 |
| 123 | HB03 | 0 | 8 | 0.02 | 0.00 | 0.00 | 0.00 | 0.98 |
| 124 | HB04 | 0 | 8 | 0.11 | 0.00 | 0.00 | 0.01 | 0.89 |
| 125 | HB05 | 0 | 8 | 0.83 | 0.02 | 0.00 | 0.00 | 0.15 |
| 126 | HB06 | 0 | 8 | 0.75 | 0.00 | 0.00 | 0.01 | 0.24 |
| 127 | HB07 | 0 | 8 | 0.82 | 0.00 | 0.00 | 0.17 | 0.01 |
| 128 | HB08 | 0 | 8 | 0.00 | 0.21 | 0.79 | 0.00 | 0.00 |
| 129 | HB09 | 0 | 8 | 0.00 | 0.20 | 0.80 | 0.00 | 0.00 |
| 130 | HB10 | 0 | 8 | 0.00 | 0.23 | 0.76 | 0.01 | 0.00 |
| 131 | HB11 | 0 | 8 | 0.00 | 0.00 | 0.99 | 0.00 | 0.00 |
| 132 | HB12 | 0 | 8 | 0.02 | 0.04 | 0.94 | 0.00 | 0.00 |
| 133 | HB13 | 0 | 8 | 0.00 | 0.00 | 1.00 | 0.00 | 0.00 |
| 134 | ZJ01 | 0 | 9 | 0.01 | 0.40 | 0.59 | 0.01 | 0.00 |
| 135 | ZJ02 | 0 | 9 | 0.00 | 0.59 | 0.40 | 0.00 | 0.00 |
| 136 | ZJ03 | 0 | 9 | 0.01 | 0.73 | 0.26 | 0.00 | 0.00 |
| 137 | ZJ04 | 0 | 9 | 0.00 | 0.97 | 0.03 | 0.00 | 0.00 |
| 138 | ZJ05 | 0 | 9 | 0.00 | 0.99 | 0.00 | 0.00 | 0.01 |
| 139 | ZJ06 | 0 | 9 | 0.00 | 0.99 | 0.01 | 0.00 | 0.00 |
| 140 | ZJ07 | 0 | 9 | 0.00 | 0.89 | 0.10 | 0.00 | 0.01 |
| 141 | ZJ08 | 0 | 9 | 0.91 | 0.00 | 0.00 | 0.00 | 0.08 |
| 142 | ZJ09 | 0 | 9 | 0.96 | 0.00 | 0.00 | 0.03 | 0.01 |
| 143 | ZJ10 | 0 | 9 | 0.32 | 0.01 | 0.00 | 0.00 | 0.66 |
| 144 | ZJ11 | 0 | 9 | 0.72 | 0.16 | 0.00 | 0.06 | 0.06 |
| 145 | ZJ12 | 0 | 9 | 0.47 | 0.01 | 0.00 | 0.00 | 0.52 |
| 146 | ZJ13 | 0 | 9 | 0.00 | 0.00 | 0.00 | 0.00 | 1.00 |
| 147 | ZJ14 | 0 | 9 | 0.02 | 0.94 | 0.04 | 0.00 | 0.00 |
| 148 | ZJ15 | 0 | 9 | 0.06 | 0.89 | 0.01 | 0.04 | 0.01 |
| 149 | ZJ16 | 0 | 9 | 0.00 | 1.00 | 0.00 | 0.00 | 0.00 |
| 150 | ZJ17 | 0 | 9 | 0.00 | 0.97 | 0.03 | 0.00 | 0.00 |
| 151 | ZJ18 | 0 | 9 | 0.00 | 0.99 | 0.01 | 0.00 | 0.00 |
| 152 | ZJ19 | 0 | 9 | 0.00 | 1.00 | 0.00 | 0.00 | 0.00 |
| 153 | ZJ20 | 0 | 9 | 0.01 | 0.99 | 0.00 | 0.00 | 0.00 |
