# Supplementary material for: Genetic diversity and population structure of Miscanthus lutarioriparius, an endemic plant of China
Source: PLoS One. 2019 Feb 1;14(2):e0211471. doi: 10.1371/journal.pone.0211471 (PMC6358086; doi:10.1371/journal.pone.0211471)
Supplement: S3 Table — (DOCX) [file pone.0211471.s004.docx]

**S3 Table Nei’s Genetic distances among populations of *Miscanthus laturaioriprius***

| **Pop** | **Pop1** | **Pop2** | **Pop3** | **Pop4** | **Pop5** | **Pop6** | **Pop7** | **Pop8** | **Pop9** |
| --- | --- | --- | --- | --- | --- | --- | --- | --- | --- |
| **Pop1** | **** |  |  |  |  |  |  |  |  |
| **Pop2** | 0.111 | **** |  |  |  |  |  |  |  |
| **Pop3** | 0.021 | 0.109 | **** |  |  |  |  |  |  |
| **Pop4** | 0.033 | 0.111 | 0.031 | **** |  |  |  |  |  |
| **Pop5** | 0.108 | 0.031 | 0.106 | 0.111 | **** |  |  |  |  |
| **Pop6** | 0.092 | 0.018 | 0.094 | 0.098 | 0.039 | **** |  |  |  |
| **Pop7** | 0.023 | 0.078 | 0.026 | 0.04 | 0.084 | 0.061 | **** |  |  |
| **Pop8** | 0.138 | 0.023 | 0.136 | 0.138 | 0.039 | 0.024 | 0.096 | **** |  |
| **Pop9** | 0.033 | 0.133 | 0.034 | 0.05 | 0.134 | 0.116 | 0.04 | 0.16 | **** |
