# Supplementary material for: Genetic diversity and population structure of Miscanthus lutarioriparius, an endemic plant of China
Source: PLoS One. 2019 Feb 1;14(2):e0211471. doi: 10.1371/journal.pone.0211471 (PMC6358086; doi:10.1371/journal.pone.0211471)
Supplement: S4 Table — (DOCX) [file pone.0211471.s005.docx]

**S4 Table Gene flow (*Nm*) among nine populations**

| Populations | Pop1 | Pop2 | Pop3 | Pop4 | Pop5 | Pop6 | Pop7 | Pop8 | Pop9 |
| --- | --- | --- | --- | --- | --- | --- | --- | --- | --- |
| Pop1 | **** |  |  |  |  |  |  |  |  |
| Pop2 | 0.72 | **** |  |  |  |  |  |  |  |
| Pop3 | 3.92 | 0.68 | **** |  |  |  |  |  |  |
| Pop4 | 6.00 | 0.94 | 6.00 | **** |  |  |  |  |  |
| Pop5 | 0.73 | 24.75 | 0.31 | 0.42 | **** |  |  |  |  |
| Pop6 | 0.61 | 12.25 | 0.61 | 0.64 | 1.32 | **** |  |  |  |
| Pop7 | 22.75 | 0.74 | 6.00 | 3.92 | 0.67 | 0.89 | **** |  |  |
| Pop8 | 0.52 | 3.92 | 0.92 | 1.12 | 8.08 | 1.54 | 0.62 | **** |  |
| Pop9 | 1.42 | 0.79 | 1.42 | 1.31 | 0.32 | 1.03 | 2.88 | 0.92 | **** |
